# Interaction between stress and the *BDNF* Val66Met polymorphism in depression: a systematic review and meta-analysis

**DOI:** 10.1186/1741-7015-12-7

**Published:** 2014-01-16

**Authors:** Georgina M Hosang, Celia Shiles, Katherine E Tansey, Peter McGuffin, Rudolf Uher

**Affiliations:** 1 Psychology Department, Goldsmiths, University of London, New Cross, London SE14 6NW, UK; 2 MRC Social, Genetic and Developmental Psychiatry Centre, Institute of Psychiatry, King’s College, London, De Crespigny Park, London SE5 8AF, UK; 3 King’s College London, Academic Centre, 2nd Floor Henriette Raphael House, Guy’s Campus, London SE1 1UL UL, UK; 4 MRC Centre for Neuropsychiatric Genetics and Genomics, Department of Psychological Medicine and Neurology, School of Medicine, Cardiff University, Cardiff CF24 4HQ, UK; 5 Department of Psychiatry, Dalhousie University, 5909 Veterans’ Memorial Lane, Halifax, Nova Scotia B3H 2E2, Canada

**Keywords:** Stress, Life events, Childhood maltreatment, Childhood adversity, Child abuse, Depression, Brain-derived neurotrophic factor, BDNF, rs6265, Gene-environment interaction

## Abstract

**Background:**

Major depression is a disabling psychiatric illness with complex origins. Life stress (childhood adversity and recent stressful events) is a robust risk factor for depression. The relationship between life stress and Val66Met polymorphism in the brain-derived neurotrophic factor (*BDNF*) gene has received much attention. The aim of the present work was to review and conduct a meta-analysis on the results from published studies examining this interaction.

**Methods:**

A literature search was conducted using PsychINFO and PubMed databases until 22 November 2013. A total of 22 studies with a pooled total of 14,233 participants met the inclusion criteria, the results of which were combined and a meta-analysis performed using the Liptak-Stouffer z-score method.

**Results:**

The results suggest that the Met allele of *BDNF* Val66Met significantly moderates the relationship between life stress and depression (*P* = 0.03). When the studies were stratified by type of environmental stressor, the evidence was stronger for an interaction with stressful life events (*P* = 0.01) and weaker for interaction of *BDNF* Val66Met with childhood adversity (*P* = 0.051).

**Conclusions:**

The interaction between *BDNF* and life stress in depression is stronger for stressful life events rather than childhood adversity. Methodological limitations of existing studies include poor measurement of life stress.

## Background

Major depression is a prevalent psychiatric illness that is associated with considerable morbidity and mortality [1]. Much research has been dedicated to uncovering its aetiology with mixed success. Environmental factors, such as childhood adversity [2,3] and stressful life events [4-6] have been associated with the onset and course of major depression. However, not everyone exposed to such adversity develops this illness or relapses [6]; this has led researchers to explore stress vulnerability factors, including interactions with genetic influences [7]. The brain-derived neurotrophic factor (*BDNF*) gene has been implicated in stress vulnerability [8]. Both variation in this gene sequence and stress exposure influence expression and intracellular trafficking of the BDNF protein. Stress is hypothesised to cause a reduction in BDNF (protein) levels in the brain (particularly in regions linked to emotion), which alters mood and may cause depression [9]. Consequently, the *BDNF* gene has been identified as a stress vulnerability candidate generating much research, which has yet to be reviewed or synthesised.

The purpose of the present work was to systematically review and perform a meta-analysis on studies that have investigated the interaction between the Val66Met polymorphism in the *BDNF* gene and life stress (childhood adversity and stressful life events) in depression.

Major depression has a lifetime prevalence of 17% [10] and is diagnosed based on the presentation of depressive symptoms (for example, low mood, sleep disturbance and suicidal behaviours) that last at least 2 weeks [1]. Depressive episodes are also experienced by individuals suffering from bipolar disorder, although the defining feature of this condition is mania (symptoms include, elation, grandiosity and irritability) [11].

Major depression is considered one of the most burdensome diseases in the world [12] and it is projected that this illness will be the second leading cause of disease burden for developed countries by 2020 [13]. Better understanding of the aetiology of this debilitating disorder is urgently required so that steps can be taken to reduce the burden on the individual, their families and society as a whole.

### Life stress and depression

There is a wealth of literature concerned with the impact of life stress and depression, with studies focusing on childhood adversity and stressful life events.

#### Childhood adversity

Childhood adversity is defined as stressful experiences that occur early in life; examples include abuse (such as sexual and physical abuse), neglect (failure of caretakers to provide the child with basic needs such as food or support), parental death and divorce. Significantly higher rates of childhood adversity are reported by adults with major depression compared to the general population [14]. Childhood adversity is also associated with worse course of depression, including lack of treatment response as well as more chronic and more frequent episodes [15].

Most studies in this area rely on retrospective reporting which is associated with various biases calling into question the validity and reliability of their findings. A recent study showed that whether childhood maltreatment is assessed prospectively (child protection database) or retrospectively (self-report), it is still significantly associated with major depression to a similar degree [16]. Moreover, Widom and colleagues [17] prospectively followed children who were abused or neglected (identified through court records), and found that as adults these individuals were at increased risk of major depression compared to matched controls.

Childhood adversity is a non-specific risk factor and has been linked to other emotional problems [18], psychiatric illnesses [16,19,20] and physical disorders [21,22]. A better understanding of the specific mechanisms that underlie the childhood adversity/depression association is needed.

#### Stressful life events

Stressful life events are described as circumstances that have a negative impact on an individual (for example, stress) that occur close to the onset or relapse of the illness [23]. Stressful events such as bereavement and divorce are common, but people’s responses to such events vary, some appear to cope well and others spiral into a depressive episode [6,24]. Although it has been shown that stressful life events cluster before a depressive episode or worsening of symptoms [4,25], there is strong evidence that one such event is sufficient to trigger an episode onset [26].

Investigating the relationship between stressful events and depression is not as straightforward as establishing whether an event occurred or not, various elements must be considered. Determining the direction of association is important, are stressful events generated by the individual and influenced by depressive symptoms (dependent events), or is it that such events occur ‘out of blue’ and trigger depressive episodes (independent events)? Establishing the event severity is essential as their impact differs between individuals and is dependent on circumstance. These key issues are addressed with the collection of contextual and biographical information best achieved using semi structured interviews [27-29]. Studies employing this method have consistently shown that severe independent events are linked to the onset, relapse and exacerbation of depressive symptoms [5,25,30], illustrating a causal relationship.

There is strong evidence for an association between life stress and depression, research now focuses on identifying stress vulnerability factors in depression including genetic factors [31,32].

### Gene/environment interactions

Genetic sensitivity to one’s environment or gene-environment interactions [33], has spurred much mental health research following Caspi and colleagues’ [31] study. Their results showed that the low expressing short allele of the serotonin-transporter-linked promoter region polymorphism (*5-HTTLPR*; a functional variant in the serotonin transporter gene) increased depressive symptoms, rates of diagnosed depression and suicidality following exposure to stressful events and childhood maltreatment [31].

Many studies that attempted to replicate these results adopted different designs using divergent samples/populations, which resulted in mixed success [34,35]. The reviews [7,28,36] and meta-analytic studies [15,37] published on this topic suggest that the interaction is strongest between *5-HTTLPR* and childhood adversity, rather than recent stressful events [37]. Life stress assessment is crucial, with studies that used objective measures or semi structured interviews rather than crude self-report questionnaires detecting the interaction with the *5-HTTLPR* variant [37]. The relationship between life stress and the serotonin transporter gene is fairly consistent for persistent and recurrent depression [14,38,39].

Other genes have been implicated in stress vulnerability including the *CRHR1*[40,41], *FKBP5*[42] and *BDNF*[43] genes. The relationship between the Val66Met variant in the *BDNF* gene and stress in depression has been widely researched and thus a systematic review and meta-analysis is warranted to synthesise the literature.

### BDNF and life stress in depression

BDNF is a protein involved in the proliferation, differentiation and survival of neuronal cells and regulation of synaptic plasticity and connectivity in the adult brain [44]. Research suggests that BDNF may also be influential in sensitivity to stress.

According to the ‘neurotrophic hypothesis’ of depression [8,45], stress reduces BDNF activity, which results in decreased function in limbic brain regions (for example, hippocampus) involved in emotion processing and cognition. This reduction in turn is posited to be associated with depressed mood; while antidepressant therapy is hypothesised to reverse this effect [9,45]. There is evidence that stress and trauma result in decreased BDNF levels in both rodents [46,47] and humans [48,49]. The findings from several meta-analyses show that lower serum BDNF levels are detected among depressed patients relative to controls but these levels are normalised with antidepressant treatment [50,51]. There is some debate surrounding the link between BDNF, antidepressants and (hippocampal) neurogenesis (see [52] for more a detailed discussion). Together the evidence presented generally supports the ‘neurotrophic hypothesis’ of depression.

A single nucleotide polymorphism (SNP) in the *BDNF* gene (that is, Val66Met; rs6265) has been shown to influence the activity of the BDNF protein. This SNP results in the substitution of valine (Val) to methionine (Met) [53]. Although the *BDNF* gene consists of several polymorphisms many are in high linkage disequilibrium and highly correlated [54,55].

The Met allele of the *BDNF* Val66Met polymorphism has been linked with reduced BDNF activity [44], memory impairment [56] and harm avoidance [57]. Individuals with at least one Met allele of this SNP and at high risk of affective disorders (co-twin diagnosed with a mood disorder) have elevated evening cortisol levels, which suggests altered stress response [58].

Transgenic mice that are Met carriers of *BDNF* Val66Met exhibit increased anxiety-related behaviours under stress conditions [59], as well as decreased BDNF levels in the hippocampus [60], in line with the ‘neurotrophic hypothesis’.

The research highlighted thus far presents a case for a significant interaction between *BDNF* Val66Met and life stress in depression. The purpose of the current paper is to systematically review and meta-analyse the literature concerned with this interaction addressing the specific question: ‘is there a significant interaction between the Met variant of *BDNF* Val66Met and life stress in depression?’

## Methods

### Data sources and search strategy

The systematic review was performed according to the ‘Preferred Reporting Items for Systematic Reviews and Meta-Analyses’ (PRISMA) guidelines [61]. The literature searches were undertaken using PsychINFO, and PubMed up until 22 November 2013 for original research studies. The search terms were synonyms for depression, life stress and the *BDNF* Val66Met polymorphism (see Table 1). These terms were searched for in the articles’ titles, keywords, tests and measures. Only papers published in the English language were considered for inclusion. The reference lists of identified studies were also searched by hand for relevant papers.

**Table 1:** Terms used for literature search

**Keyword(s)**	**Additional search terms used**
1. Depression	Depressive disorder OR major depressive disorder OR depression OR major depression OR unipolar depression OR depressive symptoms
2. Life stress	Stressful life events, OR life events OR negative life events OR adverse life events OR events OR stress OR stressors OR childhood maltreatment OR childhood adversity OR childhood stress OR child abuse OR child neglect OR adversity
3. *BDNF* Val66Met polymorphism	Brain-derived neurotrophic factor gene OR *BDNF* OR rs6265 OR Val66Met

### Eligibility criteria

The eligibility criteria were stipulated as follows: utilisation of human participants; depression (illness onset or relapse) or its symptoms were assessed; focus on *BDNF* Val66Met polymorphism (rs6265); childhood adversity and/or stressful life events (a range of types of events) were assessed; the interaction between stress and the *BDNF* Val66Met polymorphism was investigated. Studies in gross departure of Hardy-Weinberg equilibrium were excluded (for example, *P* ≤0.001).

### Data extraction

Variety of data were extracted from included studies in accordance with other gene-environment interaction reviews [37,62] (see Table 2). When publications reported results for both childhood adversity and recent stressful life events they were included in each environmental stressor group; but the results for each stressor were averaged and included in the overall analyses. The data was extracted and confirmed by two authors (GMH and KET). Where studies used multiple life stress and/or depression measures, the data yielded from interviews was included in the analyses. If the information required was not reported or unclear the authors of the original article were contacted.

**Table 2:** **Summary of studies examining the interaction between life stress and brain-derived neurotrophic factor (*BDNF*) Val66Met in depression**

**Source**	**N**	**Females,%**	**Age at study assessment (Mean or range)**	**Ancestry**	**Study design**	**Stressor**	**Life stress assessment**	**Outcome**	**Averaged one-sided *P *** **value**
Kaufman *et al*., 2006 [79]	194	51	9.3 years	Mixed	Cross-sectional	Childhood adversity	Children taken into care	Depressive symptoms	0.6765
Kim *et al*., 2007 [43]	732	59	65 years and over	East Asian	Cross-sectional	Stressful life events	Researcher-administered questionnaire	Depression diagnosis	0.000315
Wichers *et al*., 2008 [73]	464	100	18 to 46 years	European	Longitudinal twin study	Childhood adversity	Questionnaire	Depressive symptoms	0.009
Aguilera *et al*., 2009 [66]	470	55	22.9 years	European	Cross-sectional	Childhood adversity	Questionnaire	Depressive symptoms	0.637
Bukh *et al*., 2009 [82]	290	66	38.5 years (median)	European	Case only	Stressful life events	Interview	Depression onset	0.004
Gatt *et al*., 2009 [88]	374	51	36.2 years	European	Cross-sectional	Childhood adversity	Questionnaire	Depressive symptoms	0.272
Hosang *et al*., 2010 [75]	1,085	62	37.83 years	European	Case–control	Stressful life events	Researcher-administered questionnaire	Bipolar disorder relapse	0.02
Lavebratt *et al*., 2010 [68]	950	58	20 to 64 years	European	Longitudinal survey	Childhood adversity and Stressful life events	Questionnaire	Depressive diagnosis	0.5
Nederhof *et al*., 2010 [80]	1,096	53	16.13 years	European	Longitudinal study	Childhood adversity	Questionnaires and interviews	Depressive symptoms	0.3525
Carver *et al*., 2011 [76]	133	74	18.71 years	Mixed	Cross-sectional	Childhood adversity	Questionnaire	Depression diagnosis	0.01
Elzinga *et al*., 2011 [81]	1,435	69	42.2 years	European	Case only	Childhood adversity and stressful life events	Interview	Depression diagnosis and symptom severity	0.4325
Juhasz *et al*., 2011 [74]	1,269	70	34.04 years	Caucasian	Cross-sectional	Childhood adversity and stressful life events	Questionnaire	Depression diagnosis	0.13165
Chen *et al*., 2012 [69]^a^	780	51	13.6 years	Han Chinese	Longitudinal twin study	Stressful life events	Questionnaire	Depressive symptoms	0.9875
Grabe *et al*., 2012 [70]^a^	2,035	53	55.6 years	Caucasian	Case only	Childhood adversity	Questionnaire	Depressive symptoms	0.785
Herbert *et al*., 2012 [83]	279	100	36.8 years	Caucasian	Longitudinal study	Stressful life events	Interview	Depression diagnosis	0.0935
Perea *et al*., 2012 [77]	302	50	18 years	Mixed (Columbian population)	Cross-sectional	Childhood adversity and stressful life events	Questionnaire	Depressive symptoms	0.01725
Quinn *et al*., 2012 [65]	240	59	40.01 years	Unknown	Cross-sectional	Childhood adversity	Questionnaire	Melancholic subtype of depression diagnosis	0.5
Caldwell *et al*., 2013 [71]^a^	124	66	21.97 years	Mixed	Cross-sectional	Childhood adversity	Questionnaire	Depressive symptoms	0.8705
Comasco *et al*., 2013 [67]	1,393	50	17 to 18 years	European	Cross-sectional	Childhood adversity	Questionnaire	Depressive symptoms	0.251
La Greca *et al*., 2013 [78]	116	54	8.85 years	Mixed	Cross-sectional	Childhood adversity	Questionnaire	Depressive symptoms	0.167833
Jiang *et al*., 2013 [89]^a^	238	73	61.7 years	Caucasians	Cross-sectional	Stressful life events	Carer of person with dementia	Depressive symptoms	0.9805
Brown *et al*., 2013 [85]	234	100	37 years	Caucasian	Longitudinal study	Childhood adversity and stressful life events	Interview	Depressive episode onset	0.431
Total	14,233								
Average sample size	647								0.03

^a^Tested Val recessive model.

### Quality appraisal of studies

The methodological quality of included studies was evaluated against a quality checklist, derived from the STREGA (‘Strengthening the Reporting of Genetic Association Studies’) and STROBE (‘Strengthening the Reporting of Observational Studies in Epidemiology’) checklists [63,64], which have been used by other gene-environment interaction meta-analyses [37].

In accordance with current guidelines and previous reviews of gene-environment interaction studies [37], the studies were not weighted by quality scores or excluded based on low scores, but the information is presented in Additional file 1 for readers’ evaluation.

### Statistical analyses

The Liptak-Stouffer z-score method was used to combine studies at the level of significance tests with the results weighted by sample size. This approach has been used by another meta-analysis of gene-environment interactions [37]. This method involves a number of steps. First, the *P* values from the eligible studies were extracted and converted to one-tailed values, with those less than 0.50 indicating greater Met allele stress sensitivity and *P* values of greater than 0.50 corresponding to Val allele stress sensitivity in depression. Second, these *P* values were transformed into z-scores using a standard normal curve. Positive z-scores were assigned to *P* <0.50 and negative z-scores corresponded to *P* >0.50. Third, the z-scores were combined and weighted to take into account the study sample size. The following formula was used to do this:

$$\mathrm{Zw}=\frac{\sum_{i=1}^{k}w_{i}z_{i}}{\sqrt{\sum_{i=1}^{k}w_{i}^{2}}}$$

The weighting factor w_i_ corresponds to each study’s sample size, k indicates the number of total studies, and Z_i_ corresponds to the z-scores of individual studies. Z_w_, which is the outcome of this test, follows a standard normal distribution and thus related probability corresponds with a standard normal distribution table. This approach was used on the overall and stratified analyses.

Sensitivity analyses were undertaken to determine whether the results were considerably influenced by any single study; the procedure involved systematically excluding each study and recalculating the significance of the result.

To assess the potential impact of publication bias on the meta-analysis results the fail-safe N was calculated for all analyses. In line with Karg and colleagues [37], the number of studies with a *P* value equal to 0.50 and a sample size of 647 (average sample size of the studies analysed in the overall meta-analysis) were calculated that would need to be included in the weighted Liptak-Stouffer analyses to obtain a non-significant result. The ratio between the fail-safe and the actual number of studies published provides an estimation of the possibility of publication bias effecting our findings.

## Results

### Study selection

**Figure 1:**
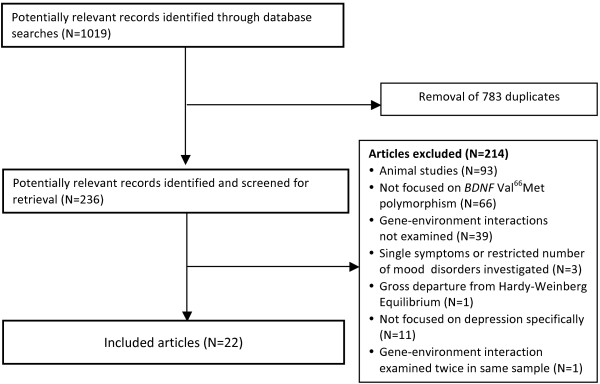
**Flow diagram of studies considered for inclusion.** This flow diagram depicts each step in the process of study selection.

A combination of the predefined search terms (depression + life stress + *BDNF* Val66Met) resulted in 1,019 hits; after duplicates were removed, 236 studies remained (see Figure 1). The abstracts of these articles were reviewed and those studies that reported on the interaction between life stress and *BDNF* Val66Met in depression were included. No existing systematic or meta-analytic reviews were found.

### Overall evidence for BDNF × stress in mood disorders

**Figure 2:**
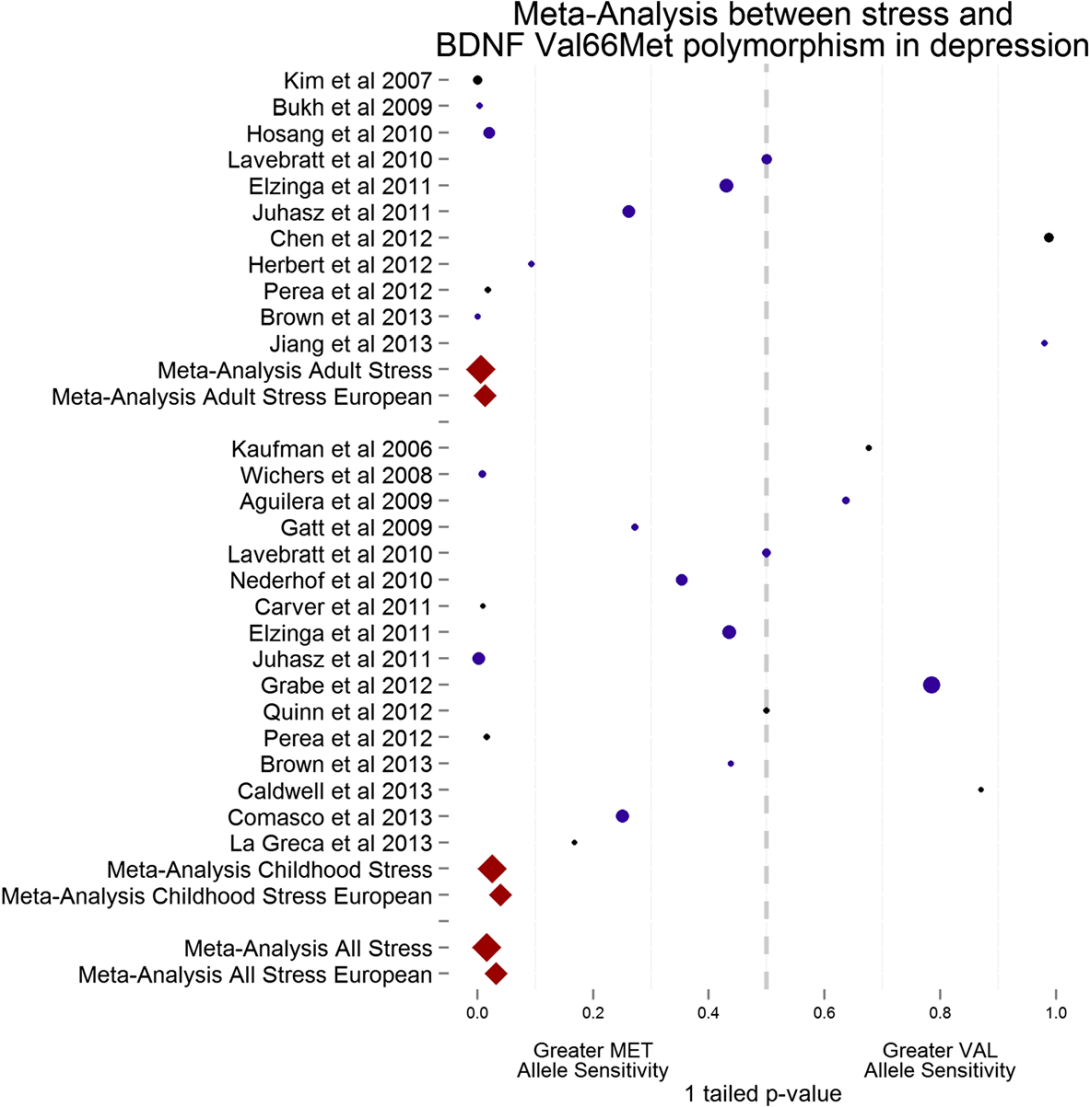
**Forest plot for included studies examining the interaction between the Met variant of the brain-derived neurotrophic factor (*BDNF*) gene and life stress in depression.**

Out of the 22 studies included, 8 reported a significant interaction between life stress (either life events or child adversity) and the Met variant of *BDNF* Val66Met in depression (see Table 2 and Figure 2).

Other studies provided partial evidence of a significant stress × *BDNF* for specific types of depression [65], childhood adversity [66,67] or detected a gender specific effect [68].

Several studies found a significant interaction between life stress and *BDNF* in depression using a Val recessive model [69-71]. The allele frequencies of the *BDNF* Val66Met polymorphism differs between ethnic groups, with the Met allele frequency highest among Asian populations [72]. Two of these studies used either a Han Chinese sample [69], or a cohort with mixed ethnicity [71], which may have contributed to the unusual finding with a Val recessive model.

### Meta-analysis result

Combining the results from the identified studies with a total of 14,233 participants showed that the interaction between the Met variant of the *BDNF* Val66Met polymorphism significantly moderates the relationship between life stress and depression (*P* = 0.03). When findings from studies that used Caucasian samples were meta-analysed separately (to account for the potential confounding effect of population stratification), the results failed to reach conventional level of significance although they were in the expected direction (*P* = 0.07).

The sensitivity analyses showed that the results remained significant after removing each study in turn (>0.004 *P* <0.049) with the exception of 4 studies [43,73-75]. In terms of publication bias, to make these results non-significant (*P* = 0.05), more than 11 unpublished or undiscovered studies with an average sample of 657 participants reporting a non-significant finding (*P* = 0.50) would need to exist. This corresponds to a fail-safe ratio of 2 studies not included in the meta-analysis for every included study.

Since there is evidence for the differential interaction effect with child adversity and stressful life events with other SNPs (for example, *5-HTTLPR*[43]), the results pertaining to the impact of these types of stressors and the *BDNF* gene were examined.

### Childhood adversity

In all, 16 studies explored the interaction between childhood adversity and *BDNF* in depression, 4 reported a significant effect [73,74,76,77] and 5 showed partial support for this interaction [65-68,78] (see Table 3).

**Table 3:** Studies included in the childhood adversity meta-analysis

**Source**	**Sample size**	**One-sided *P *** **value**
Kaufman *et al*., 2006 [79]	194	0.6765
Wichers *et al*., 2008 [73]	464	0.009
Aguilera *et al*., 2009 [66]	470	0.637
Gatt *et al*., 2009 [88]	374	0.272
Lavebratt *et al*., 2010 [68]	642	0.5
Nederhof *et al*., 2010 [80]	1,096	0.3525
Carver *et al*., 2011 [76]	133	0.01
Juhasz *et al*., 2011 [74]	1,269	0.0018
Elzinga *et al*., 2011 [81]	1,435	0.435
Grabe *et al*., 2012 [70]	2,035	0.785
Quinn *et al*., 2012 [65]	240	0.5
Perea *et al*., 2012 [77]	302	0.016
La Greca *et al*., 2013 [78]	116	0.167833
Caldwell *et al*., 2013 [71]	124	0.8705
Comasco *et al*., 2013 [67]	1,393	0.251
Brown *et al*., 2013 [85]	234	0.431
Total	10,521	
Average sample size	658	0.051

For instance, rather than investigating child adversity as one overarching construct, Aguilera and colleagues [66] focused on different types and only found a significant interaction between child sexual abuse and *BDNF*. Given the acknowledged heterogeneity in major depression [1], Quinn and associates [65] explored the impact of the child adversity-*BDNF* interaction on different subtypes of the disorder and found a significant effect for non-melancholic depression only.

The childhood adversity measures used by studies is particularly noteworthy, with all of the those reporting a significant interaction relying on questionnaires, while many of those studies not reporting an effect utilised more objective methods [79-81].

### Childhood adversity: meta-analysis results

In meta-analysing the results from included studies with a combined sample of 10,521 individuals, a trend towards a significant interaction between childhood adversity and *BDNF* in depression was detected (*P* = 0.051), but was not improved on when only those studies with Caucasian samples were analysed (*P* = 0.08).

In undertaking sensitivity analyses, the results remained non-significant after removing each study in turn (>0.052 *P* <0.27), with the exception of 6 [66,68,70,71,79,81].

### Stressful life events

The stressful events-*BDNF* interaction in depression was investigated in 11 studies using a combined sample of 7,594 individuals; 5 uncovered a significant effect (see Table 4). The majority of these studies used either researcher-administered questionnaires [43,75] or interviews [82], while the non-replicating studies relied on self-report checklists with one exception [83].

**Table 4:** Studies included in the stressful life events meta-analysis

**Source**	**Sample size**	**One-sided *P *** **value**
Kim *et al*., 2007 [43]	732	0.000315
Bukh *et al*., 2009 [82]	290	0.004
Hosang *et al*., 2010 [75]	1,085	0.02
Lavebratt *et al*., 2010 [68]	950	0.5
Juhasz *et al*., 2011 [74]	1,269	0.2615
Elzinga *et al*., 2011 [81]	1,435	0.43
Chen *et al*., 2012 [69]	780	0.9875
Perea *et al*., 2012 [77]	302	0.0185
Herbert *et al*., 2012 [83]	279	0.0935
Jiang *et al*., 2013 [89]	238	0.9805
Brown *et al*., 2013 [85]	234	0.0004
Total	7,594	
Average sample size	690	0.01

Bipolar disorder was the focus of one study [75], which found that the effect of stressful life events on episodes of bipolar depression were significantly greater in Met allele carriers (Met/Met and Val/Met) relative to those with the Val/Val genotype. However, no such pattern was reported for manic episodes [75].

### Stressful life events: meta-analysis results

A significant interaction between stressful life events and the Met variant of *BDNF* Val66Met (*P* = 0.01) was found; the results remained significant when the analyses were restricted to studies using Caucasian samples (*P* = 0.027).

Sensitivity analysis showed the *P* value remained significant with the exclusion of each study from the analysis (>0.001 *P* <0.023), except for 2 [43,75].

For this result to be non-significant (*P* = 0.05) more than 14 unpublished or unidentified studies need to exist with an average sample of 690 participants and a non-significant result (*P* = 0.50). This corresponds to a fail-safe ratio of 1 study not included for every study entered into the meta-analysis.

## Discussion

A total of 22 studies with a combined sample of 14,233 participants met the eligibility criteria, 8 of which reported a significant interaction between life stress and the Met variant of *BDNF* Val66Met. The overall meta-analysis found evidence for a significant interaction between life stress and *BDNF* in depression using a Met dominant model. When the analyses were stratified by type of stressor, the results were strongest for stressful life events while the effect of childhood adversity failed to reach conventional levels of significance.

These findings help build a lifespan model of causation for major depression focusing on several gene-stress interactions. Research shows that childhood adversity (distal and stable risk factor) significantly interacts with the short allele of the *5-HTTLPR*[37], while the findings from this meta-analysis suggest that the stressful life events (proximal and provoking factor) significantly interacts with the Met variant of *BDNF* in depression.

It has also been postulated that individuals who are genetically sensitivity to adverse experiences (for example, Met carriers) may benefit most from positive environments or even the lack of adversity, under the ‘differentially susceptibility’ hypothesis [84]. One study [85] found that the likelihood of experiencing a depressive episode was significantly reduced among individuals with at least one Met allele of *BDNF* but only in the absence of a severe stressful event. It is possible that interventions that focus on providing supportive and positive environments would be most beneficial to individuals who are genetically sensitivity to stress.

The aetiology of psychiatric disorders such as major depression is complex and is likely to involve multiple interactions between genetic variants and environmental factors. Few studies have explored the three-way interaction between childhood adversity, stressful life events and stress vulnerability genetic variants [86]. A wealth of research shows that adversity in childhood sensitises individuals to adult stressful life events increasing their risk for major depression [87]. Given the mounting evidence that genetic variants such as *BDNF* Val66Met polymorphism are important stress vulnerability factors sets the stage for future studies that should explore the childhood adversity × stressful life events × *BDNF* interaction in the context of depression.

### Child adversity

When examining the specific influence of child adversity, 4 out of 16 studies reported a significant interaction with *BDNF* Val66Met for depression, and 5 were able to provide partial support for this interaction [65-68]. However, the results from the meta-analysis of these studies provided only a trend towards a significant effect.

On closer inspection of these results it appears that the childhood adversity-*BDNF* relationship in depression may be complex, with some studies reporting a significant interaction among women and not men [68,73], and for specific subtypes of depression (non-melancholic) [65]. In addition, specific forms of childhood adversity maybe driving these associations (for example, child sexual abuse [66]). More research in this area is warranted focusing on specific forms of adversity and depression subtypes as well as investigating gender differences.

The non-replicating studies have two characteristics that are noteworthy. Firstly, some studies which failed to detect any evidence for a child adversity × *BDNF* in depression used child and adolescent cohorts [79,80]. Secondly, more reliable (for example, interviews [80,81]) and objective measures [79] of childhood adversity were adopted by these studies in comparison to those which found evidence for a gene-environment interaction who mainly used questionnaires (for example, [74,76]). There are several possible explanations for these null findings. The interaction between child adversity and *BDNF* may be developmentally sensitive and only manifests later in life (beyond adolescence). It is also possible that the *BDNF*-childhood adversity relationship in depression is a measurement artefact. Thus, future studies should assess an adult sample using interviews or objective measures of child adversity.

### Stressful life events

The results of the meta-analysis showed that the effect of stressful life events on depression is significantly moderated by the *BDNF* polymorphism. The majority of the positive studies measured stressful events using researcher-administered questionnaires or interviews while the majority of the non-replicating studies utilised self-report checklists. It is possible that more objective and sensitive approaches to measuring stressful life events are required to observe its interaction with *BDNF* in depression.

### BDNF

The *BDNF* gene consists of a number of polymorphisms which may also be relevant for gene-environment interactions in depression [55]. The majority of published studies have focused *BDNF* Val66Met, which allows for the present meta-analysis, but it would be useful for future studies to explore the relationship between other *BDNF* polymorphisms and stress in depression.

### Limitations

The studies reviewed in this article are subject to a number of limitations. Firstly, different life stress measures were employed. Questionnaires present the biggest challenge as they rely on self-report and are vulnerable to various biases such as the participant’s subjective interpretation of what counts as an event and enquire about a restricted number of events. Despite these limitations, most studies relied on self-report questionnaires, casting doubt on their results. Some studies have attempted to overcome these problems by using trained researchers to administer the questionnaires providing some improvement. More robust methods, such as life stress interviews or use of social services records of child maltreatment are more objective and combat many of the biases associated with self-report.

Secondly, the majority of included studies used Caucasian samples, which may have influenced the results. It is possible that the *BDNF* × stress in depression is only relevant to Caucasian populations, although significant interactions have been reported among Asian [43] and ethnically mixed populations [76,77]. Future studies need to recruit participants from non-Caucasian backgrounds to test the generalisability of the *BDNF* × stress in depression to other ethnic groups.

Thirdly, several included studies may have limited power due to their restricted sample sizes (for example, [65,78]). For the current study, power analyses revealed that using the pooled samples provided greater than 80% power to detect significant effects of all three interactions tested in this project (these are, (1) life stress × *BDNF*, (2) childhood adversity × *BDNF*, (3) stressful life events × *BDNF* in depression).

Finally, the statistical approach used to conduct the current meta-analysis relied on combining the *P* values of existing studies, which were weighted by sample size. This approach was adopted to maximise the number of studies that could be included, with the aim of reducing biases associated with selecting a homogeneous set of studies and to better reflect the current evidence in this field. The meta-analytic method of combining *P* values could be improved upon by using traditional approaches that focus on combining effect sizes, which benefit from providing the magnitude of an effect.

## Conclusions

A total of 22 studies with a pooled sample of 14,233 individuals were included in this review, of which 8 provided evidence in support of an interaction between life stress and the *BDNF* Val66Met polymorphism in depression. A meta-analysis provided evidence for an interaction between the Met variant of the *BDNF* Val66Met polymorphism and life stress in the causation of depression. When stratified by the type of stress, the results of the meta-analyses were only significant for stressful life events and not childhood adversity. This suggests that serotonin transporter and *BDNF* genes may confer sensitivity to early and late environmental factors respectively and play complementary yet distinct roles in the depression aetiology.

## Competing interests

GMH has provided sponsored talks for Bristol-Myers Squibb.

## Authors’ contributions

GMH was responsible for drafting the manuscript and collation of the data for analysis. KET undertook the analyses for the study, contributed to collation and checking the data. CS conducted the initial literature search and collation of data. RU and PMcG contributed to the design of the study and interpretation of the results. All authors were involved in drafting the manuscript and have given final approval of the version to be published.

## Pre-publication history

The pre-publication history for this paper can be accessed here:

http://www.biomedcentral.com/1741-7015/12/7/prepub

## Supplementary Material

Additional file 1: Table S1 Quality assessment of brain-derived neurotrophic factor (BDNF), life stress and depression studies [43,65-71,73-83,85,88,89]. Click here for file

## References

[B1] McGuffin P Murray RM, Kendler KS, McGuffin P, Wessely S, Castle D Affective disorders Essential Psychiatry 2008 Cambridge, UK: Cambridge University Press 250 283

[B2] Alloy LB Abramson LY Smith JM Gibb BE Neeren AM Role of parenting and maltreatment histories in unipolar and bipolar mood disorders: mediation by cognitive vulnerability to depression Clin Child Fam Psychol Rev 2006 9 23 64 10.1007/s10567-006-0002-4 16718583

[B3] Fisher H Hosang GM Childhood maltreatment and bipolar disorder: a critical review of the evidence Mind Brain J Psychiatr 2010 1 75 85

[B4] Hosang GM Korszun A Jones L Jones I Gray JM Gunasinghe CM McGuffin P Farmer AE Adverse life event reporting and worst illness episodes in unipolar and bipolar affective disorders: measuring environmental risk for genetic research Psychol Med 2010 40 1829 1837 10.1017/S003329170999225X 20132580

[B5] Farmer AE McGuffin P Humiliation, loss and other types of life events and difficulties: a comparison of depressed subjects, healthy controls and their siblings Psychol Med 2003 33 1169 1175 10.1017/S0033291703008419 14580071

[B6] Hammen C Stress and depression Annu Rev Clin Psychol 2005 1 293 319 10.1146/annurev.clinpsy.1.102803.143938 17716090

[B7] Caspi A Hariri AR Holmes A Uher R Moffitt TE Genetic sensitivity to the environment: the case of the serotonin transporter gene and its implications for studying complex diseases and traits Am J Psychiatry 2010 167 509 527 10.1176/appi.ajp.2010.09101452 20231323 PMC2943341

[B8] Groves JO Is it time to reassess the BDNF hypothesis of depression? Mol Psychiatry 2007 12 1079 1088 10.1038/sj.mp.4002075 17700574

[B9] Stein DJ Daniels WMU Savitz J Harvey BH Brain-derived neurotrophic factor: the neurotrophin hypothesis of psychopathology CNS Spectr 2008 13 945 949 19037180 10.1017/s1092852900013997

[B10] Kessler RC Chiu WT Demler O Merikangas KR Walters EE Prevalence, severity, and comorbidity of 12-month DSM-IV disorders in the National Comorbidity Survey Replication Arch Gen Psychiatry 2005 62 617 627 10.1001/archpsyc.62.6.617 15939839 PMC2847357

[B11] Miklowitz DJ Johnson SL The psychopathology and treatment of bipolar disorder Annu Rev Clin Psychol 2006 2 199 235 10.1146/annurev.clinpsy.2.022305.095332 17716069 PMC2813703

[B12] Lopez AD Mathers CD Ezzati M Jamison DT Murray CJ Global and regional burden of disease and risk factors, 2001: systematic analysis of population health data Lancet 2006 367 1747 1757 10.1016/S0140-6736(06)68770-9 16731270

[B13] Menken M Munsat TL Toole JF The global burden of disease study: implications for neurology Arch Neurol 2000 57 418 420 10.1001/archneur.57.3.418 10714674

[B14] Fisher HL Cohen-Woods S Hosang GM Korszun A Owen M Craddock N Craig IW Farmer AE McGuffin P Uher R Interaction between specific forms of childhood maltreatment and the serotonin transporter gene (5-HTT) in recurrent depressive disorder J Affect Disord 2013 145 136 141 10.1016/j.jad.2012.05.032 22840631 PMC3825663

[B15] Nanni V Uher R Danese A Childhood maltreatment predicts unfavorable course of illness and treatment outcome in depression: a meta-analysis Am J Psychiatry 2012 169 141 151 22420036 10.1176/appi.ajp.2011.11020335

[B16] Scott KM McLaughlin KA Smith DA Ellis PM Childhood maltreatment and DSM-IV adult mental disorders: comparison of prospective and retrospective findings Br J Psychiatry 2012 200 469 475 10.1192/bjp.bp.111.103267 22661679 PMC3365274

[B17] Widom CS DuMont K Czaja SJ A prospective investigation of major depressive disorder and comorbidity in abused and neglected children grown up Arch Gen Psychiatry 2007 64 49 56 10.1001/archpsyc.64.1.49 17199054

[B18] Agnafors S Comasco E Bladh M Sydsjo G Dekeyser L Oreland L Svedin CG Effect of gene, environment and maternal depressive symptoms on pre-adolescence behavior problems - a longitudinal study Child Adolesc Psychiatry Ment Health 2013 7 10 10.1186/1753-2000-7-10 23518193 PMC3615948

[B19] Morgan C Fisher H Environment and schizophrenia: environmental factors in schizophrenia: childhood trauma-a critical review Schizophr Bull 2007 33 3 10 17105965 10.1093/schbul/sbl053 PMC2632300

[B20] Kessler RC McLaughlin KA Green JG Gruber MJ Sampson NA Zaslavsky AM Aguilar-Gaxiola S Alhamzawi AO Alonso J Angermeyer M Benjet C Bromet E Chatterji S de Girolamo G Demyttenaere K Fayyad J Florescu S Gal G Gureje O Haro JM Hu CY Karam EG Kawakami N Lee S Lépine JP Ormel J Posada-Villa J Sagar R Tsang A Ustün TB Childhood adversities and adult psychopathology in the WHO World Mental Health Surveys Br J Psychiatry 2010 197 378 385 10.1192/bjp.bp.110.080499 21037215 PMC2966503

[B21] Hosang GM Johnson SL Kiecolt-Glaser J Di Gregorio MP Lambert DR Bechtel MA Hearne DW Herron JB Glaser R Gender specific association of child abuse and adult cardiovascular disease in a sample of patients with basal cell carcinoma Child Abuse Negl 2013 37 374 379 10.1016/j.chiabu.2012.09.018 23347911 PMC3845899

[B22] Scott KM Von Korff M Angermeyer MC Benjet C Bruffaerts R de Girolamo G Haro JM Lépine JP Ormel J Posada-Villa J Tachimori H Kessler RC Association of childhood adversities and early-onset mental disorders with adult-onset chronic physical conditions Arch Gen Psychiatry 2011 68 838 844 10.1001/archgenpsychiatry.2011.77 21810647 PMC3402030

[B23] Ezquiaga E Ayuso Gutierrez JL Garcia Lopez A Psychosocial factors and episode number in depression J Affect Disord 1987 12 135 138 10.1016/0165-0327(87)90005-X 2955004

[B24] Hosang GM Korszun A Jones L Jones I McGuffin P Farmer AE Life-event specificity: bipolar disorder compared with unipolar depression Br J Psychiatry 2012 201 458 465 10.1192/bjp.bp.112.111047 23137729

[B25] Hosang GM Uher R Maughan B McGuffin P Farmer AE The role of loss and danger events in symptom exacerbation in bipolar disorder J Psychiatr Res 2012 46 1584 1589 10.1016/j.jpsychires.2012.07.009 22868047

[B26] Brown GW Harris TO Social Origins of Depression. A Study of Psychiatric Disorder in Women 1978 London, UK: Routledge

[B27] Uher R McGuffin P The moderation by the serotonin transporter gene of environmental adversity in the aetiology of mental illness: review and methodological analysis Mol Psychiatry 2008 13 131 146 10.1038/sj.mp.4002067 17700575

[B28] Uher R McGuffin P The moderation by the serotonin transporter gene of environmental adversity in the etiology of depression: 2009 update Mol Psychiatry 2010 15 18 22 10.1038/mp.2009.123 20029411

[B29] Monroe SM Reid MW Gene-environment interactions in depression research: genetic polymorphisms and life-stress polyprocedures Psychol Sci 2008 19 947 956 10.1111/j.1467-9280.2008.02181.x 19000200

[B30] Maciejewski PK Prigerson HG Mazure CM Sex differences in event-related risk for major depression Psychol Med 2001 31 593 604 11352362 10.1017/s0033291701003877

[B31] Caspi A Sugden K Moffitt TE Taylor A Craig IW Harrington H McClay J Mill J Martin J Braithwaite A Poulton R Influence of life stress on depression: moderation by a polymorphism in the 5-HTT gene Science 2003 301 386 389 10.1126/science.1083968 12869766

[B32] Uher R Forum: The case for gene-environment interactions in psychiatry Curr Opin Psychiatry 2008 21 318 321 10.1097/YCO.0b013e328306a7b9 18520730

[B33] Moffitt TE Caspi A Rutter M Strategy for investigating interactions between measured genes and measured environments Arch Gen Psychiatry 2005 62 473 481 10.1001/archpsyc.62.5.473 15867100

[B34] Eley TC Sugden K Corsico A Gregory AM Sham P McGuffin P Plomin R Craig IW Gene-environment interaction analysis of serotonin system markers with adolescent depression Mol Psychiatry 2004 9 908 915 10.1038/sj.mp.4001546 15241435

[B35] Surtees PG Wainwright NW Willis-Owen SA Luben R Day NE Flint J Social adversity, the serotonin transporter (5-HTTLPR) polymorphism and major depressive disorder Biol Psychiatry 2006 59 224 229 10.1016/j.biopsych.2005.07.014 16154545

[B36] Brown GW Harris TO Depression and the serotonin transporter 5-HTTLPR polymorphism: a review and a hypothesis concerning gene-environment interaction J Affect Disord 2008 111 1 12 10.1016/j.jad.2008.04.009 18534686

[B37] Karg K Burmeister M Shedden K Sen S The serotonin transporter promoter variant (5-HTTLPR), stress, and depression meta-analysis revisited: evidence of genetic moderation Arch Gen Psychiatry 2011 68 444 454 10.1001/archgenpsychiatry.2010.189 21199959 PMC3740203

[B38] Brown GW Ban M Craig TK Harris TO Herbert J Uher R Serotonin transporter length polymorphism, childhood maltreatment, and chronic depression: a specific gene-environment interaction Depress Anxiety 2013 30 5 13 10.1002/da.21982 22847957

[B39] Uher R Caspi A Houts R Sugden K Williams B Poulton R Moffitt TE Serotonin transporter gene moderates childhood maltreatment’s effects on persistent but not single-episode depression: replications and implications for resolving inconsistent results J Affect Disord 2011 135 56 65 10.1016/j.jad.2011.03.010 21439648 PMC3752793

[B40] Polanczyk G Caspi A Williams B Price TS Danese A Sugden K Uher R Poulton R Moffitt TE Protective effect of CRHR1 gene variants on the development of adult depression following childhood maltreatment: replication and extension Arch Gen Psychiatry 2009 66 978 985 10.1001/archgenpsychiatry.2009.114 19736354 PMC3750953

[B41] Bradley RG Binder EB Epstein MP Tang Y Nair HP Liu W Gillespie CF Berg T Evces M Newport DJ Stowe ZN Heim CM Nemeroff CB Schwartz A Cubells JF Ressler KJ Influence of child abuse on adult depression: moderation by the corticotropin-releasing hormone receptor gene Arch Gen Psychiatry 2008 65 190 200 10.1001/archgenpsychiatry.2007.26 18250257 PMC2443704

[B42] Zimmermann P Bruckl T Nocon A Pfister H Binder EB Uhr M Lieb R Moffitt TE Caspi A Holsboer F Ising M Interaction of FKBP5 gene variants and adverse life events in predicting depression onset: results from a 10-year prospective community study Am J Psychiatry 2011 168 1107 1116 21865530 10.1176/appi.ajp.2011.10111577 PMC3856576

[B43] Kim JM Stewart R Kim SW Yang SJ Shin IS Kim YH Yoon JS Interactions between life stressors and susceptibility genes (5-HTTLPR and BDNF) on depression in Korean elders Biol Psychiatry 2007 62 423 428 10.1016/j.biopsych.2006.11.020 17482146

[B44] Post RM Role of BDNF in bipolar and unipolar disorder: clinical and theoretical implications J Psychiatr Res 2007 41 979 990 10.1016/j.jpsychires.2006.09.009 17239400

[B45] Duman RS Monteggia LM A neurotrophic model for stress-related mood disorders Biol Psychiatry 2006 59 1116 1127 10.1016/j.biopsych.2006.02.013 16631126

[B46] Nibuya M Takahashi M Russell DS Duman RS Repeated stress increases catalytic TrkB mRNA in rat hippocampus Neurosci Lett 1999 267 81 84 10.1016/S0304-3940(99)00335-3 10400217

[B47] Roceri M Cirulli F Pessina C Peretto P Racagni G Riva MA Postnatal repeated maternal deprivation produces age-dependent changes of brain-derived neurotrophic factor expression in selected rat brain regions Biol Psychiatry 2004 55 708 714 10.1016/j.biopsych.2003.12.011 15038999

[B48] Kauer-Sant’Anna M Tramontina J Andreazza AC Cereser K da Costa S Santin A Yatham LN Kapczinski F Traumatic life events in bipolar disorder: impact on BDNF levels and psychopathology Bipolar Disord 2007 9 128 135 10.1111/j.1399-5618.2007.00478.x 17543031

[B49] Grassi-Oliveira R Stein LM Lopes RP Teixeira AL Bauer ME Low plasma brain-derived neurotrophic factor and childhood physical neglect are associated with verbal memory impairment in major depression - a preliminary report Biol Psychiatry 2008 64 281 285 10.1016/j.biopsych.2008.02.023 18406398

[B50] Molendijk ML Spinhoven P Polak M Bus BA Penninx BW Elzinga BM Serum BDNF concentrations as peripheral manifestations of depression: evidence from a systematic review and meta-analyses on 179 associations (N=9484) Mol Psychiatryin press 10.1038/mp.2013.105 23958957

[B51] Sen S Duman R Sanacora G Serum brain-derived neurotrophic factor, depression, and antidepressant medications: meta-analyses and implications Biol Psychiatry 2008 64 527 532 10.1016/j.biopsych.2008.05.005 18571629 PMC2597158

[B52] Jiang C Salton SR The Role of Neurotrophins in Major Depressive Disorder Transl Neurosci 2013 4 46 58 10.2478/s13380-013-0103-8 23691270 PMC3656715

[B53] Rybakowski JK BDNF gene: functional Val66Met polymorphism in mood disorders and schizophrenia Pharmacogenomics 2008 9 1589 1593 10.2217/14622416.9.11.1589 19018714

[B54] Uher R Huezo-Diaz P Perroud N Smith R Rietschel M Mors O Hauser J Maier W Kozel D Henigsberg N Barreto M Placentino A Dernovsek MZ Schulze TG Kalember P Zobel A Czerski PM Larsen ER Souery D Giovannini C Gray JM Lewis CM Farmer A Aitchison KJ McGuffin P Craig I Genetic predictors of response to antidepressants in the GENDEP project Pharmacogenomics J 2009 9 225 233 10.1038/tpj.2009.12 19365399

[B55] Liu L Foroud T Xuei X Berrettini W Byerley W Coryell W El-Mallakh R Gershon ES Kelsoe JR Lawson WB MacKinnon DF McInnis M McMahon FJ Murphy DL Rice J Scheftner W Zandi PP Lohoff FW Niculescu AB Meyer ET Edenberg HJ Nurnberger JI Jr Evidence of association between brain-derived neurotrophic factor gene and bipolar disorder Psychiatr Genet 2008 18 267 274 10.1097/YPG.0b013e3283060f59 19018231 PMC2653694

[B56] Egan MF Kojima M Callicott JH Goldberg TE Kolachana BS Bertolino A Zaitsev E Gold B Goldman D Dean M Lu B Weinberger DR The BDNF val66met polymorphism affects activity-dependent secretion of BDNF and human memory and hippocampal function Cell 2003 112 257 269 10.1016/S0092-8674(03)00035-7 12553913

[B57] Jiang X Xu K Hoberman J Tian F Marko AJ Waheed JF Harris CR Marini AM Enoch MA Lipsky RH BDNF variation and mood disorders: a novel functional promoter polymorphism and Val66Met are associated with anxiety but have opposing effects Neuropsychopharmacology 2005 30 1353 1361 15770238 10.1038/sj.npp.1300703

[B58] Vinberg M Trajkovska V Bennike B Knorr U Knudsen GM Kessing LV The BDNF Val66Met polymorphism: relation to familiar risk of affective disorder, BDNF levels and salivary cortisol Psychoneuroendocrinology 2009 34 1380 1389 10.1016/j.psyneuen.2009.04.014 19473771

[B59] Chen ZY Jing D Bath KG Ieraci A Khan T Siao CJ Herrera DG Toth M Yang C McEwen BS Hempstead BL Lee FS Genetic variant BDNF (Val66Met) polymorphism alters anxiety-related behavior Science 2006 314 140 143 10.1126/science.1129663 17023662 PMC1880880

[B60] Murakami S Imbe H Morikawa Y Kubo C Senba E Chronic stress, as well as acute stress, reduces BDNF mRNA expression in the rat hippocampus but less robustly Neurosci Res 2005 53 129 139 10.1016/j.neures.2005.06.008 16024125

[B61] Liberati A Altman DG Tetzlaff J Mulrow C Gotzsche PC Ioannidis JP Clarke M Devereaux PJ Kleijnen J Moher D The PRISMA statement for reporting systematic reviews and meta-analyses of studies that evaluate healthcare interventions: explanation and elaboration BMJ 2009 339 b2700 10.1136/bmj.b2700 19622552 PMC2714672

[B62] Munafo MR Durrant C Lewis G Flint J Gene X environment interactions at the serotonin transporter locus Biol Psychiatry 2009 65 211 219 10.1016/j.biopsych.2008.06.009 18691701

[B63] Little J Higgins JP Ioannidis JP Moher D Gagnon F von Elm E Khoury MJ Cohen B Davey-Smith G Grimshaw J Scheet P Gwinn M Williamson RE Zou GY Hutchings K Johnson CY Tait V Wiens M Golding J van Duijn C McLaughlin J Paterson A Wells G Fortier I Freedman M Zecevic M King R Infante-Rivard C Stewart A Birkett N Strengthening the reporting of genetic association studies (STREGA): an extension of the STROBE Statement Hum Genet 2009 125 131 151 10.1007/s00439-008-0592-7 19184668

[B64] von Elm E Altman DG Egger M Pocock SJ Gotzsche PC Vandenbroucke JP Initiative S The Strengthening the Reporting of Observational Studies in Epidemiology (STROBE) statement: guidelines for reporting observational studies Lancet 2007 370 1453 1457 10.1016/S0140-6736(07)61602-X 18064739

[B65] Quinn CR Dobson-Stone C Outhred T Harris A Kemp AH The contribution of BDNF and 5-HTT polymorphisms and early life stress to the heterogeneity of major depressive disorder: a preliminary study Aust N Z J Psychiatry 2012 46 55 63 10.1177/0004867411430878 22247094

[B66] Aguilera M Arias B Wichers M Barrantes-Vidal N Moya J Villa H van Os J Ibanez MI Ruiperez MA Ortet G Fananas L Early adversity and 5-HTT/BDNF genes: new evidence of gene-environment interactions on depressive symptoms in a general population Psychol Med 2009 39 1425 1432 10.1017/S0033291709005248 19215635

[B67] Comasco E Aslund C Oreland L Nilsson KW Three-way interaction effect of 5-HTTLPR, BDNF Val66Met, and childhood adversity on depression: a replication study Eur Neuropsychopharmacol 2013 23 1300 1306 10.1016/j.euroneuro.2013.01.010 23481907

[B68] Lavebratt C Aberg E Sjoholm LK Forsell Y Variations in FKBP5 and BDNF genes are suggestively associated with depression in a Swedish population-based cohort J Affect Disord 2010 125 249 255 10.1016/j.jad.2010.02.113 20226536

[B69] Chen J Li X McGue M Interacting effect of BDNF Val66Met polymorphism and stressful life events on adolescent depression Genes Brain Behav 2012 11 958 965 10.1111/j.1601-183X.2012.00843.x 22931410

[B70] Grabe HJ Schwahn C Mahler J Appel K Schulz A Spitzer C Fenske K Barnow S Freyberger HJ Teumer A Petersmann A Biffar R Rosskopf D John U Völzke H Genetic epistasis between the brain-derived neurotrophic factor Val66Met polymorphism and the 5-HTT promoter polymorphism moderates the susceptibility to depressive disorders after childhood abuse Prog Neuropsychopharmacol Biol Psychiatry 2012 36 264 270 10.1016/j.pnpbp.2011.09.010 21996278

[B71] Caldwell W McInnis OA McQuaid RJ Liu G Stead JD Anisman H Hayley S The role of the Val66Met polymorphism of the brain derived neurotrophic factor gene in coping strategies relevant to depressive symptoms PLoS One 2013 8 e65547 10.1371/journal.pone.0065547 23824678 PMC3688808

[B72] Petryshen TL Sabeti PC Aldinger KA Fry B Fan JB Schaffner SF Waggoner SG Tahl AR Sklar P Population genetic study of the brain-derived neurotrophic factor (BDNF) gene Mol Psychiatry 2010 15 810 815 10.1038/mp.2009.24 19255578 PMC2888876

[B73] Wichers M Kenis G Jacobs N Mengelers R Derom C Vlietinck R van Os J The BDNF Val(66)Met x 5-HTTLPR x child adversity interaction and depressive symptoms: An attempt at replication Am J Med Genet B Neuropsychiatr Genet 2008 147B 120 123 10.1002/ajmg.b.30576 17579366

[B74] Juhasz G Dunham JS McKie S Thomas E Downey D Chase D Lloyd-Williams K Toth ZG Platt H Mekli K Payton A Elliott R Williams SR Anderson IM Deakin JF The CREB1-BDNF-NTRK2 pathway in depression: multiple gene-cognition-environment interactions Biol Psychiatry 2011 69 762 771 10.1016/j.biopsych.2010.11.019 21215389

[B75] Hosang GM Uher R Keers R Cohen-Woods S Craig I Korszun A Perry J Tozzi F Muglia P McGuffin P Farmer AE Stressful life events and the brain-derived neurotrophic factor gene in bipolar disorder J Affect Disord 2010 125 345 349 10.1016/j.jad.2010.01.071 20172611

[B76] Carver CS Johnson SL Joormann J Lemoult J Cuccaro ML Childhood adversity interacts separately with 5-HTTLPR and BDNF to predict lifetime depression diagnosis J Affect Disord 2011 132 89 93 10.1016/j.jad.2011.02.001 21420735

[B77] Perea CS Paternina AC Gomez Y Lattig MC Negative affectivity moderated by BDNF and stress response J Affect Disord 2012 136 767 774 10.1016/j.jad.2011.09.043 22044630

[B78] La Greca AM Lai BS Joormann J Auslander BB Short MA Children’s risk and resilience following a natural disaster: Genetic vulnerability, posttraumatic stress, and depression J Affect Disord 2013 151 860 867 10.1016/j.jad.2013.07.024 24035489

[B79] Kaufman J Yang BZ Douglas-Palumberi H Grasso D Lipschitz D Houshyar S Krystal JH Gelernter J Brain-derived neurotrophic factor-5-HTTLPR gene interactions and environmental modifiers of depression in children Biol Psychiatry 2006 59 673 680 10.1016/j.biopsych.2005.10.026 16458264

[B80] Nederhof E Bouma EM Oldehinkel AJ Ormel J Interaction between childhood adversity, brain-derived neurotrophic factor val/met and serotonin transporter promoter polymorphism on depression: the TRAILS study Biol Psychiatry 2010 68 209 212 10.1016/j.biopsych.2010.04.006 20553751

[B81] Elzinga BM Molendijk ML Oude Voshaar RC Bus BA Prickaerts J Spinhoven P Penninx BJ The impact of childhood abuse and recent stress on serum brain-derived neurotrophic factor and the moderating role of BDNF Val66Met Psychopharmacology (Berl) 2011 214 319 328 10.1007/s00213-010-1961-1 20703451 PMC3045516

[B82] Bukh JD Bock C Vinberg M Werge T Gether U Vedel Kessing L Interaction between genetic polymorphisms and stressful life events in first episode depression J Affect Disord 2009 119 107 115 10.1016/j.jad.2009.02.023 19339052

[B83] Herbert J Ban M Brown GW Harris TO Ogilvie A Uher R Craig TK Interaction between the BDNF gene Val/66/Met polymorphism and morning cortisol levels as a predictor of depression in adult women Br J Psychiatry 2012 201 313 319 10.1192/bjp.bp.111.107037 22844024 PMC3461447

[B84] Belsky J Pluess M Beyond diathesis stress: differential susceptibility to environmental influences Psychol Bull 2009 135 885 908 19883141 10.1037/a0017376

[B85] Brown GW Craig TK Harris TO Herbert J Hodgson K Tansey KE Uher R Functional polymorphism in the Brain-derived neurotrophic factor gene interacts with stressful life events but not childhood maltreatment in the etiology of depression Depress Anxiety DOI: 10.1002/da.22221 10.1002/da.22221 24338983

[B86] Power RA Lecky-Thompson L Fisher HL Cohen-Woods S Hosang GM Uher R Powell-Smith G Keers R Tropeano M Korszun A Jones L Jones I Owen MJ Craddock N Craig IW Farmer AE McGuffin P The interaction between child maltreatment, adult stressful life events and the 5-HTTLPR in major depression J Psychiatr Res 2013 47 1032 1035 10.1016/j.jpsychires.2013.03.017 23618376

[B87] Nederhof E Schmidt MV Mismatch or cumulative stress: toward an integrated hypothesis of programming effects Physiol Behav 2012 106 691 700 10.1016/j.physbeh.2011.12.008 22210393

[B88] Gatt JM Nemeroff CB Dobson-Stone C Paul RH Bryant RA Schofield PR Gordon E Kemp AH Williams LM Interactions between BDNF Val66Met polymorphism and early life stress predict brain and arousal pathways to syndromal depression and anxiety Mol Psychiatry 2009 14 681 695 10.1038/mp.2008.143 19153574

[B89] Jiang R Brummett BH Babyak MA Siegler IC Williams RB Brain-derived neurotrophic factor (BDNF) Val66Met and adulthood chronic stress interact to affect depressive symptoms J Psychiatr Res 2013 47 233 239 10.1016/j.jpsychires.2012.10.009 23140671 PMC3605893

