# Supplementary material for: Interaction between stress and the BDNF Val66Met polymorphism in depression: a systematic review and meta-analysis
Source: BMC Med. 2014 Jan 16;12:7. doi: 10.1186/1741-7015-12-7 (PMC3912923; doi:10.1186/1741-7015-12-7)
Supplement: Additional file 1: Table S1 — Quality assessment of brain-derived neurotrophic factor (BDNF), life stress and depression studies [43,65-71,73-83,85,88,89]. [file 1741-7015-12-7-S1.pdf]

Supplementary table: Quality Assessment of BDNF, life stress and depression studies

Study	Introduction	Methods						Results		
	Objectives & hypothesis clearly stated	Clear eligibility criteria for participants	Clear definition for each variable	Statistical Methods replicable	Assessment of HWE	Assessment of ethnicity	Mixed ethnicity addressed statistically	Sufficient descriptive data (eg. age, sex etc)	Genotype frequencies stated	Sample in HWE
Kaufman et al 2007 [79]	+	+	+	+	-	+	+	+	+	N/A
Kim et al 2007 [43]	+	-	+	+	+	Not stated	Not stated	-	+	+
Wichers et al 2008 [73]	+	-	+	+	+	+	Unnecessary	+	+	+
Bukh et al 2009 [82]	+	+	+	+	+	+	Unnecessary	+	+	+
Gatt et al 2009 [88]	+	+	+	+	+	+	Unnecessary	+	+	+
Aguilera et al 2009 [66]	+	+	+	+	+	+	Unnecessary	+	+	+
Hosang et al 2010 [75]	+	+	+	+	+	+	Unnecessary	+	+	+
Lavebratt et al 2010 [68]	-	-	+	+	+	Swedish Nationals	Unnecessary	+	+	Results not reported
Nederhof et al 2010 [80]	+	-	+	+	-	+	Unnecessary	+	+	N/A
Carver et al 2011 [76]	+	-	+	+	+	+	+	+	+	+

Elzinga et al 2011 [81]	+	+	+	+	+	+	Unnecessary	+	+	+
Juhasz et al 2011 [74]	+	+	+	+	+	+	Unnecessary	+	+	+
Chen et al 2012 [69]	+	+	+	+	+	+	Unnecessary	+	+	+
Grabe et al 2012 [70]	+	+	+	+	+	+	Unnecessary	+	+	+
Herbert et al 2012 [83]	+	+	+	+	+	+	Unnecessary	+	+	+
Quinn et al 2012 [65]	+	+	+	+	+	-	-	+	+	+
Perea et al 2012 [77]	+	-	+	+	+	-	-	+	+	+
La Greca et al 2013 [78]	+	-	+	+	-	+	+	+	+	N/A
Caldwell et al 2013 [71]	+	-	+	+	-	+	-	+	+	N/A
Comasco et al 2013 [67]	+	+	+	+	-	+	Unnecessary	+	+	N/A
Jiang et al 2013 [89]	+	+	+	+	+	+	Unnecessary	+	+	+
Brown et al 2013 [85]	+	+	+	+	+	+	+	+	+	+

Abbreviations: HWE, Hardy-Weinberg Equilibrium; BDNF, Brain-derived neurotrophic factor.
